# Burden, Clinical Characteristics, Risk Factors, and Seasonality of Adenovirus 40/41 Diarrhea in Children in Eight Low-Resource Settings

**DOI:** 10.1093/ofid/ofac241

**Published:** 2022-05-13

**Authors:** Godfrey Guga, Sarah Elwood, Caroline Kimathi, Gagandeep Kang, Margaret N Kosek, Aldo A M Lima, Pascal O Bessong, Amidou Samie, Rashidul Haque, Jose Paulo Leite, Ladaporn Bodhidatta, Najeeha Iqbal, Nicola Page, Ireen Kiwelu, Zulfiqar A Bhutta, Tahmeed Ahmed, Jie Liu, Elizabeth T Rogawski McQuade, Eric Houpt, James A Platts-Mills, Estomih R Mduma

**Affiliations:** Haydom Global Health Research Centre, Haydom, Tanzania; Division of Infectious Diseases and International Health, University of Virginia, Charlottesville, Virginia, USA; Haydom Global Health Research Centre, Haydom, Tanzania; Christian Medical College, Vellore, India; Division of Infectious Diseases and International Health, University of Virginia, Charlottesville, Virginia, USA; Asociación Benéfica PRISMA, Iquitos, Peru; Federal University of Ceara, Fortaleza, Brazil; University of Venda, Thohoyandou, South Africa; University of Venda, Thohoyandou, South Africa; International Centre for Diarrhoeal Disease Research, Bangladesh, Dhaka, Bangladesh; Fundação Oswaldo Cruz (Fiocruz), Rio de Janeiro, Brazil; Armed Forces Research Institute of Medical Sciences (AFRIMS), Bangkok, Thailand; Aga Khan University, Karachi, Pakistan; National Institute for Communicable Diseases, Johannesburg, South Africa; Kilimanjaro Clinical Research Institute, Moshi, Tanzania; Aga Khan University, Karachi, Pakistan; International Centre for Diarrhoeal Disease Research, Bangladesh, Dhaka, Bangladesh; Division of Infectious Diseases and International Health, University of Virginia, Charlottesville, Virginia, USA; School of Public Health, Qingdao University, Shandong, China; Rollins School of Public Health, Emory University, Atlanta, Georgia, USA; Division of Infectious Diseases and International Health, University of Virginia, Charlottesville, Virginia, USA; Division of Infectious Diseases and International Health, University of Virginia, Charlottesville, Virginia, USA; Haydom Global Health Research Centre, Haydom, Tanzania

**Keywords:** adenovirus, children, diarrhea, qPCR, seasonality

## Abstract

**Background:**

The application of molecular diagnostics has identified enteric group adenovirus serotypes 40 and 41 as important causes of diarrhea in children. However, many aspects of the epidemiology of adenovirus 40/41 diarrhea have not been described.

**Methods:**

We used data from the 8-site Etiology, Risk Factors, and Interactions of Enteric Infections and Malnutrition and the Consequences for Child Health and Development Project birth cohort study to describe site- and age-specific incidence, risk factors, clinical characteristics, and seasonality.

**Results:**

The incidence of adenovirus 40/41 diarrhea was substantially higher by quantitative polymerase chain reaction than enzyme immunoassay and peaked at ∼30 episodes per 100 child-years in children aged 7–15 months, with substantial variation in incidence between sites. A significant burden was also seen in children 0–6 months of age, higher than other viral etiologies with the exception of rotavirus. Children with adenovirus 40/41 diarrhea were more likely to have a fever than children with norovirus, sapovirus, and astrovirus (adjusted odds ratio [aOR], 1.62; 95% CI, 1.16–2.26) but less likely than children with rotavirus (aOR, 0.66; 95% CI, 0.49–0.91). Exclusive breastfeeding was strongly protective against adenovirus 40/41 diarrhea (hazard ratio, 0.64; 95% CI, 0.48–0.85), but no other risk factors were identified. The seasonality of adenovirus 40/41 diarrhea varied substantially between sites and did not have clear associations with seasonal variations in temperature or rainfall.

**Conclusions:**

This study supports the situation of adenovirus 40/41 as a pathogen of substantial importance, especially in infants. Fever was a distinguishing characteristic in comparison to other nonrotavirus viral etiologies, and promotion of exclusive breastfeeding may reduce the high observed burden in the first 6 months of life.

Diarrhea is a significant public health problem, in particular in children in low-resource settings [1]. While the introduction of rotavirus vaccines has led to important reductions in disease [2], further work is needed to understand and control other leading etiologies. The enteric group adenovirus serotypes 40 and 41 were first convincingly established as agents of diarrhea after development of antigen-based detection methods in the 1980s [3], and the majority of the subsequent 3 decades of work on the burden, epidemiology, and clinical characteristics of this pathogen utilized an enzyme immunoassay (EIA) [4–8]. In these studies, it was generally identified as the cause of <5% of pediatric diarrhea. The recent application of quantitative molecular diagnostics to etiologic studies of diarrhea has substantially increased estimates of the burden of diarrhea due to enteric adenoviruses [9, 10]. In a re-analysis of the Global Enterics Multicenter Study using quantitative polymerase chain reaction (qPCR) for a broad range of pathogens, adenovirus 40/41 had the second highest burden of diarrhea in infants after rotavirus [10]. Similarly, adenovirus 40/41 had the second highest attributable burden of severe diarrhea in children <2 years of age in a rotavirus vaccine clinical trial conducted at 3 sites in India [11] and was associated with a high burden of diarrhea requiring hospitalization, especially in Asia, in testing of samples from the Global Rotavirus Surveillance Network [12].

We have previously reported pathogen-specific burdens of diarrhea in the Etiology, Risk Factors, and Interactions of Enteric Infections and Malnutrition and the Consequences for Child Health and Development Project (MAL-ED), a birth cohort study performed at 8 sites in South America, Sub-Saharan Africa, and Asia [13]. Using quantitative molecular diagnostics, adenovirus 40/41 had the fourth highest burden of diarrhea of any severity identified by active surveillance and the second highest in infants [9], a marked increase over prior estimates from the same study based on enzyme immunoassay [4]. In the present study, we sought to characterize the epidemiology of the enteric adenoviruses as detected by qPCR in the MAL-ED cohorts in greater detail, including a comparison of incidence with EIA detection, age- and site-specific burdens, clinical characteristics, risk factors, and seasonality.

## METHODS

### Study Design and Procedures

The MAL-ED study design and methods are described in detail elsewhere [13]. In brief, the study was conducted at 8 sites in Dhaka, Bangladesh, Fortaleza, Brazil, Vellore, India, Bhaktapur, Nepal, Nausharo Feroze, Pakistan, Loreto, Peru, Venda, South Africa, and Haydom, Tanzania, from November 2009 to February 2014. Infants <17 days old were enrolled and followed with twice-weekly home visits until they reached 2 years of age. During visits, field workers identified diarrhea episodes, defined as 3 or more loose stools in 24 hours or visible blood in stool and recorded breastfeeding practices. Clinical characteristics of diarrhea episodes were determined by maternal report. Socioeconomic and demographic data were collected semi-annually, including household income, maternal education, household demographics, hygiene practices, and water and sanitation indicators, as defined by World Health Organization guidelines [14]. Collection of a single stool sample was attempted during each diarrheal episode as well as monthly in the absence of diarrhea. Monthly average rainfall and temperature data for each site were extracted from an online database [15].

### Laboratory Testing

Standardized protocols were used for detection of enteropathogens at each site laboratory, and details of both the original enteropathogen diagnostics and the quantitative PCR re-analysis have been previously published [4, 9]. Briefly, adenovirus was orignally detected from diarrheal stools as well as nondiarrheal stools collected at months 1–12, 15, 18, 21, and 24 using an enzyme immunoassay (ProSpecT, Remel, Lenexa, KS, USA), with no characterization of the subgroup of enteric adenoviruses. Raw stool aliquots were stored at −80°C until extraction of total nucleic acid, with incorporation of external controls to monitor the efficiency of extraction and amplification. Using customized TaqMan Array Cards, adenoviruses were detected by quantitative PCR targeting the Hexon gene, and serotypes 40/41 were also specifically detected by quantitative PCR targeting the Fiber gene [10]. Although a cycle threshold of 35 was considered the analytical limit of detection, to increase the clinical specificity for these analyses we used a cycle threshold cutoff of 30 to define infection across all pathogens.

### Data Analysis

We used a previously developed approach to attribute diarrhea etiology by first modeling the association between pathogen detection and diarrhea. We then calculated population attributable fractions (AFs) by site using detection by both enzyme immunoassay and qPCR for adenovirus 40/41. We also calculated age-stratified AFs using qPCR detection of the 5 major viral etiologies of diarrhea identified in MAL-ED [1, 2]. At the level of each individual episode, pathogen-attributable diarrhea was defined as diarrhea with a pathogen- and episode-specific attributable fraction (AFe) >0.5, as previously described [9]. To compare the clinical characteristics of adenovirus-attributable diarrhea episodes with those of other diarrhea attributable to other viruses, we fit a logistic regression model for each characteristic to determine the odds of attribution to adenovirus rather than other viral etiologies, adjusting for site and the AFe for *Shigella* and enterotoxigenic *E. coli*, the bacterial enteropathogens with the highest attributable burden in this study [9].

To model risk factors for adenovirus 40/41 infection, we used the Prentice, Williams, & Peterson total time extension of the Cox model to allow for multiple detections per child over the course of the study without requiring that events are independent of each other to obtain hazard ratios [16]. Study site and other baseline risk factors were included in the model based on plausibility. Exclusive breastfeeding status varied over the course of follow-up time in the model. The same models were also fit using an outcome of infection with any nonadenovirus viral enteropathogen.

To evaluate for seasonal trends, we focused on infection, rather than attributable diarrhea, because this allowed for greater precision, with the assumption that seasonality is driven by exposure, rather than susceptibility to clinical illness. We used Poisson harmonic regressions to describe seasonality both averaged over a calendar year and over the multiple-year period of follow-up. For both models, linear, quadratic, and cubic terms for the median age of the cohort at that week of the study (*w*) were included to control for the effect of an aging cohort on the incidence of detection and the terms $sin\left(\frac{2\pi w}{53}\right),\;cosin\left(\frac{2\pi w}{53}\right)\;,\;sin\left(\frac{4\pi w}{53}\right)$, and $cosin\left(\frac{4\pi w}{53}\right)\;$ to capture the seasonal variation [17]. The final model was selected based on model fit as determined by the Akake Information Criterion. Likelihood ratio tests compared nested models with and without seasonal terms to determine if the seasonal model better fit the data. We also used a likelihood ratio test on nested models with and without year of infection to interrogate whether incidence varied from year to year. All statistical analysis was done in R, version 3.5.2 (Foundation for Statistical Computing). The risk factor analysis used the ‘survival’ package, version 2.43-3.

## RESULTS

Of the 42 488 samples with qPCR results for at least 1 pathogen from 1715 children in the MAL-ED cohort, 41 333 samples had valid results for adenovirus 40/41 and were included in the analysis. Adenoviruses were detected by EIA in 264 of 6573 diarrheal stools (4.0%) and 642 of 22 745 nondiarrheal stools (2.8%) that were tested by this method. Adenoviruses, including nonenteric adenoviruses, were detected by qPCR in 1253 of 6742 (18.6%) diarrheal stools, while adenovirus 40/41 was detected in 698 of 6748 diarrheal stools (10.3%) and 1509 of 34 585 (4.4%) nondiarrheal surveillance stools and was an attributable etiology for 411 diarrhea episodes (6.1%) (Supplementary Tables 1 and 2). Almost half (199; 48.4%) of these diarrhea episodes were also attributed to at least 1 other pathogen. Of those, 77 (38.7%) were also attributable to *Shigella*, 56 (28.1%) to rotavirus, 49 (24.6%) to heat-stable toxin-producing enterotoxigenic *E. coli*, 29 (14.6%) to sapovirus, 11 (5.5%) to norovirus, and 10 (5%) to astrovirus.

**Figure 1.**
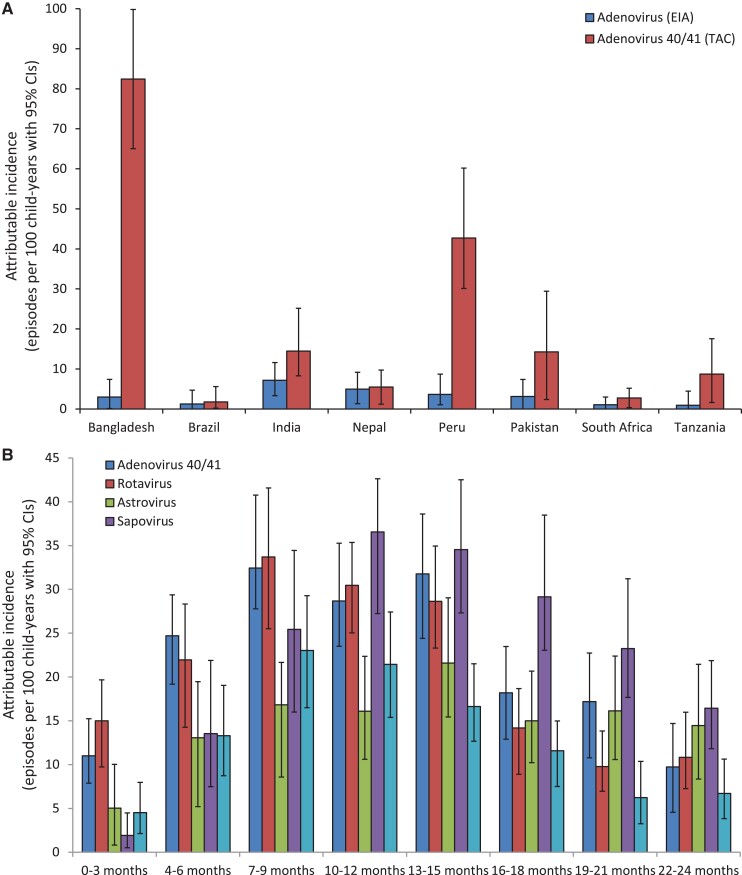
Attributable incidence of adenovirus 40/41 diarrhea in children 0–24 months of age in the MAL-ED birth cohort study, estimated by EIA and qPCR (A) and by qPCR for 3-month age groups (B). Abbreviations: EIA, enzyme immunoassay; MAL-ED, Etiology, Risk Factors, and Interactions of Enteric Infections and Malnutrition and the Consequences for Child Health and Development Project; qPCR, quantitative polymerase chain reaction.

Adenovirus 40/41–attributable diarrhea incidence increased substantially when estimated using qPCR in comparison with EIA and varied substantially between sites, with the highest incidence seen in the Dhaka, Bangladesh, site and the majority of incidence seen in Dhaka, Bangladesh, and Loreto, Peru (Figure 1*A*). The age-specific attributable incidence of adenovirus 40/41 was similar to rotavirus and notably higher than for other viral etiologies in the first 6 months of life but still peaked between 7 and 15 months of age, with an attributable incidence of ∼30 episodes per 100 child-years in that age group (Figure 1B). Children aged 0–6 months (adjusted odds ratio [aOR], 3.58; 95% CI, 2.08–6.20) and 7–12 months (aOR, 1.64; 95% CI, 1.06–2.56) had a higher odds of adenovirus 40/41 diarrhea compared with other nonrotavirus viral causes of gastroenteritis (Table 1).

**Table 1.** Association Between Clinical Characteristics of Diarrhea and Attribution to Adenovirus 40/41 vs Rotavirus and Other Viruses (Norovirus, Sapovirus, and Astrovirus)

Clinical Characteristics	Adenovirus 40/41	Rotavirus	Other Viruses^a^	Adenovirus 40/41 vs Rotavirus,	Adenovirus 40/41 vs Other Viruses,^a^
	(n = 411)	(n = 559)	(n = 1122)	aOR (95% CI)^b^	aOR (95% CI)^b^
Age					
0–6 mo	83 (20.2)	112 (20.0)	105 (9.4)	1.25 (0.74–2.13)	3.58 (2.08–6.20)
7–12 mo	153 (37.2)	228 (40.8)	394 (35.1)	1.02 (0.64–1.64)	1.64 (1.06–2.56)
13–18 mo	116 (28.2)	143 (25.6)	397 (35.4)	1.20 (0.74–1.95)	1.41 (0.91–2.19)
19–24 mo	59 (14.4)	76 (13.6)	226 (20.1)	Referent	Referent
Presence of blood in stool	17 (4.1)	12 (2.1)	27 (2.4)	1.82 (0.77–4.56)	1.34 (0.62–2.87)
Subjective fever	107 (26.0)	215 (38.5)	281 (25.0)	0.66 (0.49–0.91)	1.62 (1.16–2.26)
Duration of diarrhea episode, d	3.9 (2.8)	4.2 (3.1)	4.2 (3.4)	1.03 (0.97–1.09)	1.05 (0.99–1.10)
Maximum No. of loose stools	5.7 (2.5)	6.4 (3.0)	5.4 (2.1)	0.94 (0.89–0.99)	1.05 (0.99–1.12)
Presence of vomiting	137 (33.3)	272 (48.7)	331 (29.5)	0.66 (0.49–0.89)	1.34 (0.99–1.81)
Severe diarrhea (score >6) [4]	61 (14.8)	154 (27.6)	150 (13.4)	0.62 (0.43–0.90)	1.43 (0.94–2.17)
Dehydration (any)	33 (8.0)	94 (16.8)	106 (9.4)	0.60 (0.37–0.95)	1.22 (0.72–2.01)
Loss of appetite	134 (38.0)	144 (31.5)	286 (32.3)	0.84 (0.61–1.17)	0.87 (0.63–1.21)

Values shown are No. (%) for dichotomous variables and mean (SD) for continuous variables. Abbreviation: aOR, adjusted odds ratio.

a Astrovirus, norovirus, and sapovirus.

b Adjusted for site and attribution to *Shigella* or enterotoxigenic *E. coli*.

Fever was associated with adenovirus 40/41–attributable diarrhea in comparison with other nonrotavirus viral causes (aOR, 1.62; 95% CI, 1.16–2.26). Children with vomiting, a higher number of loose stools, severe diarrhea, and dehydration were more likely to have diarrhea attributable to rotavirus than adenovirus 40/41, and these characteristics did not differentiate adenovirus 40/41 and other nonrotavirus causes. Females had a slightly decreased risk of infection with nonadenovirus viral enteropathogens, with an 11% reduction in hazard compared with males, where there was no association between sex and adenovirus 40/41 infections. Similarly, improved sanitation and drinking water source were protective against diarrhea due to other viruses, but not adenovirus 40/41. Exclusive breastfeeding, which occurred for a median (interquartile range) of 66 (27–123) days, was protective against both adenovirus 40/41 (hazard ratio [HR], 0.64; 95% CI, 0.48–0.85) and other viral infections (HR, 0.80; 95% CI, 0.70–0.91) (Table 2). No other risk factors were identified.

**Table 2.** Association Between Sociodemographic Factors and Infection With Adenovirus 40/41 and Other Viruses

	Adenovirus 40/41,	Other Viruses,^a^
	Hazard Ratio (95% CI)^b^	Hazard Ratio (95% CI)^b^
Female sex	1.08 (0.98–1.18)	0.94 (0.89–0.99)
Current exclusive breastfeeding	0.64 (0.48–0.85)	0.80 (0.70–0.91)
Improved sanitation (WHO criteria)	1.03 (0.90–1.19)	0.88 (0.81–0.96)
Maternal education (>6 y completed)	1.01 (0.91–1.12)	0.95 (0.89–1.02)
<3 children at home	0.99 (0.89–1.09)	0.97 (0.91–1.03)
Improved drinking water source	1.20 (0.95–1.53)	0.89 (0.79–1.00)
Treated water	0.98 (0.86–1.12)	0.92 (0.85–1.01)
Wash hands before eating	0.99 (0.87–1.13)	0.97 (0.90–1.05)
Wash hands after defecating	0.95 (0.84–1.07)	0.99 (0.92–1.06)
Enrollment weight-for-age Z score	0.99 (0.94–1.04)	0.98 (0.95–1.01)

Abbreviation: WHO, World Health Organization.

a Astrovirus, norovirus, rotavirus, or sapovirus.

b Adjusted for study site and stool type (diarrheal vs nondiarrhea surveillance).

**Figure 2.**
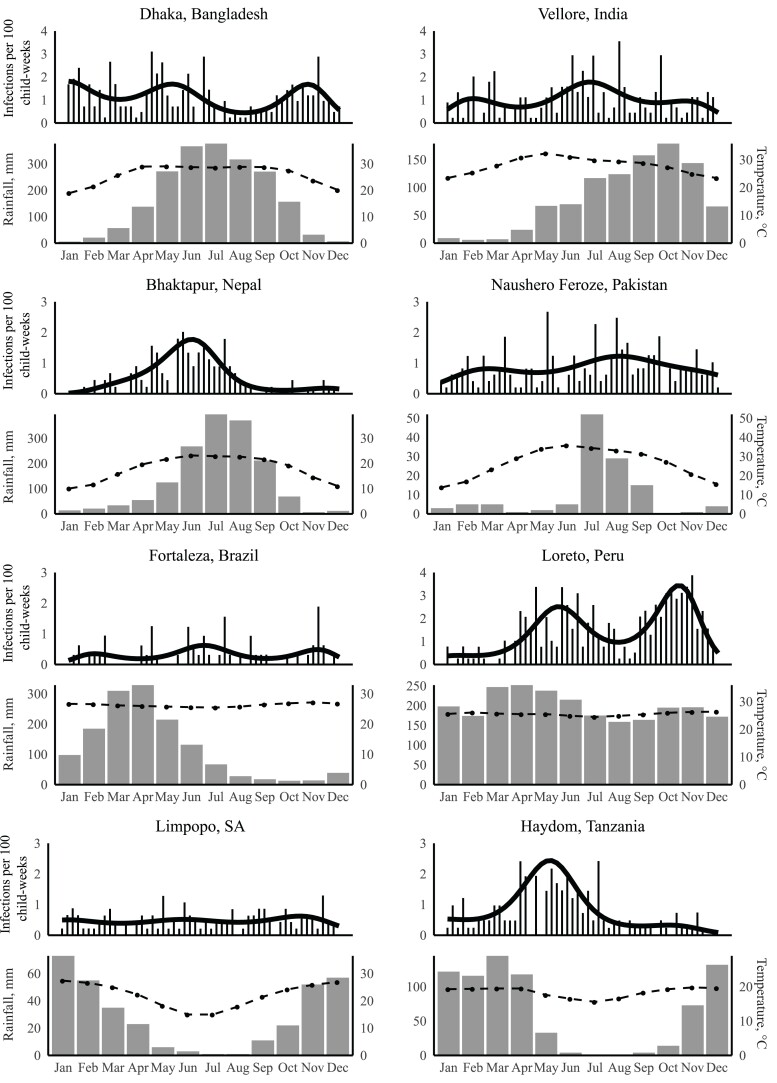
Annual incidence of adenovirus 40/41 infection by calendar month, monthly average temperature, and rainfall for each site. The solid line represents the predicted incidence, the gray bars represent monthly rainfall, and the dotted line represents temperature.

**Figure 3.**
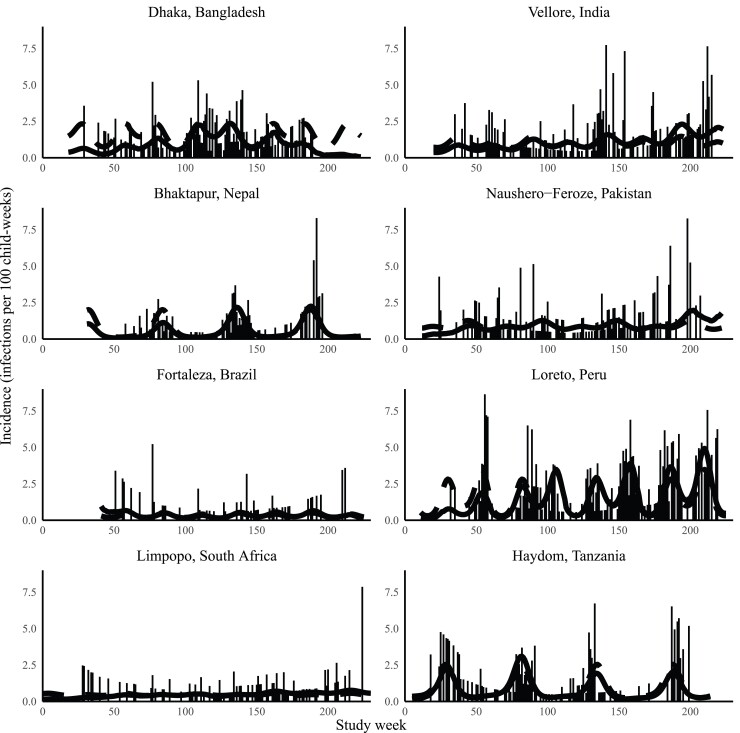
Incidence of adenovirus 40/41 infection over the course of the study by site and study month. The dotted line represents the predicted incidence of adenovirus 40/41 infection if the cohort age was held constant at 365 days. Week 0 starts on November 10, 2009, and week 230 starts on March 5, 2014.

There was substantial variation in the degree of seasonality seen for adenovirus 40/41 infection by site. Four sites exhibited statistically significant seasonal differences: Bangladesh (*P* = .001), Nepal (*P* < .001), Peru (*P* < .001), and Tanzania (*P* < .001). Hayom, Tanzania, and Bhaktapur, Nepal, had striking monophasic seasonality. Dhaka, Bangladesh, and Loreto, Peru, had evidence of multiple peaks in infection incidence (Figure 2). There was no clear relationship between rainfall or average temperature and incidence of adenovirus 40/41 infection. The seasonal peaks were evident across multiple study years; age-adjusted incidence was statistically significantly different year to year only in Nepal (*P* < .001), Peru (*P* < .001), and Tanzania (*P* < .001) (Figure 3).

## DISCUSSION

In this multisite, community-based birth cohort study utilizing quantitative molecular diagnostics for pathogen detection, we identified a substantial burden of adenovirus 40/41 infection and attributable diarrhea. While adenovirus 40/41 diarrhea incidence peaked between 7 and 15 months of age, there was also a substantially higher incidence in young infants than with most viruses other than rotavirus. There was also significant variation in incidence between sites—with a strikingly high incidence in the Bangladesh and Peru sites. For the adenovirus EIA used in the orignal MAL-ED study, both poor sensitivity and specificity likely contributed to the low attribution of adenovirus as an etiology of diarrhea in the original analysis [4]. The use of a targeted, quantitative assay for adenovirus 40/41 diarrhea revealed a substantially higher, if heterogenous, burden.

Significant variation in adenovirus 40/41 diarrhea burden has been previously observed in multisite etiologic studies of diarrhea, including the Global Enteric Multicentre Study (GEMS), in which the adenovirus 40/41–attributable fraction in infants varied from 7.2% in Pakistan to 22.4% in India [10], and qPCR testing of samples from the Global Rotavirus Surveillance Network, in which the attributable fraction of hospitalized diarrhea for adenovirus 40/41 ranged from 2.7% in the Region for the Americas to 8.7% in the Western Pacific Region [12]. Although we did not observe significant year-to-year variation during this study, longitudinal surveillance could help us understand the dynamics of adenovirus 40/41 incidence. Also striking was the frequent presence of coinfections, including with *Shigella*, rotavirus, and enterotoxigenic *E. coli*, consistent the high force of infection with these pathogens in these settings.

Like rotavirus, children with adenovirus 40/41 diarrhea were more likely to have a fever reported by caregivers than children with viral etiologies other than rotavirus and adenovirus 40/41, but other clinical characteristics of adenovirus 40/41 diarrhea were similar to other agents of viral gastroenteritis and less severe than rotavirus. We have previously reported an increased risk of dehydration in rotavirus and adenovirus 40/41 diarrhea, but this was in comparison with diarrhea without an identified infectious etiology, rather than diarrhea attributable to other viruses [9]. Although adenovirus 40/41 diarrhea was not seen to be more severe than diarrhea due to other nonrotavirus viruses, the relatively high incidence in the youngest children may mean that it is associated with more mortality and morbidity, especially in children with poor access to care.

Exclusive breastfeeding was clearly protective against adenovirus 40/41 diarrhea as well as other viral diarrhea. Given the significant burden of adenovirus 40/41 observed even in the first 6 months of life, it is possible that increasing the relatively short duration of exclusive breastfeeding would significantly reduce this burden. The absence of clearly definable individual risk factors is consistent with a recent analysis of the Bangladesh GEMS site [18]. Interventional studies of improved sanitation and hygiene have had variable efficacy to reduce carriage of viral enteropathogens, with a study in Bangladesh demonstrating a reduction in adenovirus 40/41, norovirus, and sapovirus [19] but a similar study in Zimbabwe showing no benefit [20]. The substantial variation in incidence between sites in the present study might implicate community- or country-level risk factors, for example, sanitation, population density, or climate.

The incidence of adenovirus 40/41 infection was strongly seasonal at some sites, but not others. As opposed to rotavirus, where incidence peaked during the colder and drier season in this study [21], and in contrast to a prior single-site study from Kenya [22], there was no clear or consistent relationship between adenovirus 40/41 infection and these climatic factors. Age-adjusted incidence was also consistent across multiple study years, suggesting that the observed high burden was endemic.

This study had several important limitations. First, in the absence of assays that specifically target non-40/41 adenoviruses, we could not evaluate the role of other serotypes as agents of diarrhea. Several reports have suggested that some nonenteric adenovirus serotypes may be associated with gastroenteritis, including serotypes 3, 9, 10, and 12 [23–25], but this has not been comprehensively evaluated in children in low-resource settings [26]. Similarly, we could not distinguish between types 40 and 41 to describe type-specific burden, clinical characteristics, risk factors, and seasonality. It is possible that these research sites, some of which have conducted community-based research for many years, are not representative. Finally, the clinical characteristics were based on maternal report in this community-based study. The inclusion of multiple geographically diverse sites, the large sample size, the use of a closed system for quantitative PCR with a standardized protocol across all labs, and the community-based design with a short recall window that allowed for identification of all episodes of diarrhea were clear strengths of this study.

In conclusion, we describe a substantial burden of adenovirus 40/41 diarrhea starting early in infancy across a diverse group of low-resource settings, implicating adenovirus 40/41 as a pathogen of substantial importance especially in infants and in the era after widespread introduction of rotavirus vaccines. Further characterization of the associated mortality and morbidity would help to establish the relative importance of disease prevention efforts focused on this pathogen.

## Supplementary Material

ofac241_Supplementary_Data

## Supplementary Data

Supplementary materials are available at *Open Forum Infectious Diseases* online. Consisting of data provided by the authors to benefit the reader, the posted materials are not copyedited and are the sole responsibility of the authors, so questions or comments should be addressed to the corresponding author.

## References

[1] GBD 2016 Diarrhoeal Disease Collaborators . Estimates of the global, regional, and national morbidity, mortality, and aetiologies of diarrhoea in 195 countries: a systematic analysis for the Global Burden of Disease Study 2016. Lancet Infect Dis 2018; 18:1211–28. 30243583 10.1016/S1473-3099(18)30362-1 PMC6202444

[2] Aliabadi N , Antoni S, Mwenda J, et al Global impact of rotavirus vaccine introduction on rotavirus hospitalisations among children under 5 years of age, 2008–2016: findings from the Global Rotavirus Surveillance Network. Lancet Glob Health 2019; 7:e893–903. 31200889 10.1016/S2214-109X(19)30207-4 PMC7336990

[3] Johansson M , Uhnoo I, Kidd A, et al Direct identification of enteric adenovirus, a candidate new serotype associated with infantile gastroenteritis. J Clin Microbiol 1980; 12:95–100. 6158526 10.1128/jcm.12.1.95-100.1980 PMC273528

[4] Platts-Mills JA , Babji S, Bodhidatta L, et al Pathogen-specific burdens of community diarrhoea in developing countries: a multisite birth cohort study (MAL-ED). Lancet Glob Health 2015; 3:e564–75. 26202075 10.1016/S2214-109X(15)00151-5 PMC7328884

[5] Kotloff K , Nataro J, Blackwelder W, et al Burden and aetiology of diarrhoeal disease in infants and young children in developing countries (the Global Enterice Multicenter Study, GEMS): a prospective, case-control study. Lancet 2013; 382:209–22. 23680352 10.1016/S0140-6736(13)60844-2

[6] Li L , Phan TG, Nguyen TA, et al Molecular epidemiology of adenovirus infection among pediatric population with diarrhea in Asia. Microbiol Immunol 2015; 49:121–8. 10.1111/j.1348-0421.2005.tb03711.x 15722597

[7] Jarecki-Kahn K , Tzipori SR, Unicomb LE. Enteric adenovirus infection among infants with diarrhea in rural Bangladesh. J Clin Microbiol 1993; 31:484–9. 8458940 10.1128/jcm.31.3.484-489.1993 PMC262806

[8] Kotloff KL , Losonsky GA, Morris JG, Wasserman S, Singh-Naz N, Levine MM. Enteric adenovirus infection and childhood diarrhea: an epidemiologic study in three clinical settings. Pediatrics 1989; 84:219–25. 2546121

[9] Platts-Mills JA , Liu J, Rogawski ET, et al Use of quantitative molecular diagnostic methods to assess the aetiology, burden, and clinical characteristics of diarrhoea in children in low-resource settings: a reanalysis of the MAL-ED cohort study. Lancet Glob Health 2018; 6:e1309–18. 30287127 10.1016/S2214-109X(18)30349-8 PMC6227251

[10] Liu J , Platts-Mills JA, Juma J, et al Use of quantitative molecular diagnostic methods to identify causes of diarrhoea in children: a reanalysis of the GEMS case-control study. Lancet 2016; 388:1291–301. 27673470 10.1016/S0140-6736(16)31529-X PMC5471845

[11] Praharaj I , Platts-Mills JA, Taneja S, et al Diarrheal etiology and impact of coinfections on rotavirus vaccine efficacy estimates in a clinical trial of a monovalent human-bovine (116E) oral rotavirus vaccine, Rotavac, India. Clin Infect Dis 2019; 69:243–250. 30335135 10.1093/cid/ciy896 PMC6603264

[12] Operario D , Platts-Mills JA, Nadan S, et al Etiology of severe acute watery diarrhea in children in the global rotavirus surveillance network using quantitative polymerase chain reaction. J Infect Dis 2017; 216:220–7. 28838152 10.1093/infdis/jix294 PMC5853801

[13] MAL-ED Network Investigators . The MAL-ED study: a multinational and multidisciplinary approach to understand the relationship between enteric pathogens, malnutrition, gut physiology, physical growth, cognitive development, and immune responses in infants and children up to 2 years of age in resource-poor environments. Clin Infect Dis 2014; 59(Suppl 4):S193–206. 25305287 10.1093/cid/ciu653

[14] WHO/UNICEF Joint Monitoring Programme for Water Supply and Sanitation . Progress on drinking water and sanitation: 2012 update. Available at: https://washdata.org/report/jmp-2012-report. Accessed 26 February 2022.

[15] Climate-Data.org. Climate data. Available at: https://en.climate-data.org/. Accessed 26 February 2022.

[16] Ozga AK , Kieser M, Rauch G. A systematic comparison of recurrent event models for application to composite endpoints. BMC Med Res Methodol 2018; 18:2. 29301487 10.1186/s12874-017-0462-x PMC5755224

[17] Stolwijk AM , Straatman H, Zielhuis GA. Studying seasonality by using sine and cosine functions in regression analysis. J Epidemiol Community Health 1999; 53:235–8. 10396550 10.1136/jech.53.4.235 PMC1756865

[18] Bray AE , Ahmed S, Das SK, et al Viral pathogen-specific clinical and demographic characteristics of children with moderate-to-severe diarrhea in rural Bangladesh. Am J Trop Med Hyg 2019; 101:304–9. 31264563 10.4269/ajtmh.19-0152 PMC6685579

[19] Grembi JA , Lin A, Karim MA, et al Effect of water, sanitation, handwashing and nutrition interventions on enteropathogens in children 14 months old; a cluster-randomized controlled trial in rural Bangladesh. J Infect Dis. 2020. [Online ahead of print]. 10.1093/infdis/jiaa549 PMC9891429 32861214

[20] McQuade ETR , Platts-Mills JA, Gratz J, et al Impact of water quality, sanitation, handwashing, and nutritional interventions on enteric infections in rural Zimbabwe: the Sanitation Hygiene Infant Nutrition Efficacy (SHINE) trial. J Infect Dis 2020; 221:1379–86. 31004129 10.1093/infdis/jiz179 PMC7325799

[21] Colston JM , Ahmed AM, Soofi SB, et al Seasonality and within-subject clustering of rotavirus infections in an eight-site birth cohort study. Epidemiol Infect 2018; 146:688–97. 29534766 10.1017/S0950268818000304 PMC9134355

[22] Lambisia AW , Onchaga S, Murunga N, et al Epidemiological trends of five common diarrhea-associated enteric viruses pre- and post-rotavirus vaccine introduction in coastal Kenya. Pathogens 2020; 9:660. 10.3390/pathogens9080660 PMC7459961 32824245

[23] Qiu F-Z , Shen X-X, Li G-X, et al Adenovirus associated with acute diarrhea: a case-control study. BMC Infect Dis 2018; 18:450. 30176819 10.1186/s12879-018-3340-1 PMC6122197

[24] Portes SAR , Volotao EM, Rocha MS, et al A non-enteric adenovirus A12 gastroenteritis outbreak in Rio de Janeiro, Brazil. Mem Inst Oswaldo Cruz 2016; 111:403–6. 27223654 10.1590/0074-02760160030 PMC4909040

[25] Dey SK , Shimizu H, Phan TG, et al Molecular epidemiology of adenovirus infection among infants and children with acute gastroenteritis in Dhaka City, Bangladesh. Infect Genet Evol 2009; 9:518–22. 19460318 10.1016/j.meegid.2009.02.001

[26] Lee B , Damon CF, Platts-Mills JA. Pediatric acute gastroenteritis associated with adenovirus 40/41 in low-income and middle-income countries. Curr Opin Infect Dis 2020; 33:398–403. 32773498 10.1097/QCO.0000000000000663 PMC8286627

