# Supplementary material for: Burden, Clinical Characteristics, Risk Factors, and Seasonality of Adenovirus 40/41 Diarrhea in Children in Eight Low-Resource Settings
Source: Open Forum Infect Dis. 2022 May 13;9(7):ofac241. doi: 10.1093/ofid/ofac241 (PMC9277636; doi:10.1093/ofid/ofac241)
Supplement: ofac241_Supplementary_Data [file ofac241_supplementary_data.docx]

Supplementary material

[Table S1. Detection of adenovirus 40/41 by qPCR in diarrheal and non-diarrheal (monthly) stools by site and overall, using cycle threshold < 35. 2](#_Toc101542853)

[Table S2. Detection of adenovirus 40/41 by qPCR in diarrheal and non-diarrheal (monthly) stools by site and overall, using cycle threshold < 30. 3](#_Toc101542854)

Table S1. Detection of adenovirus 40/41 by qPCR in diarrheal and non-diarrheal (monthly) stools by site and overall, using cycle threshold < 35.

| **Site** | **Stool type** | **Negative**  **(N=35221)** | **Positive**  **(N=6112)** | **Total**  **(N=41333)** |
| --- | --- | --- | --- | --- |
| Overall | Diarrhea | 5033 (74.6%) | 1715 (25.4%) | 6748 |
|  | Monthly | 30188 (87.3%) | 4397 (12.7%) | 34585 |
|  |  |  |  |  |
| BG | Diarrhea | 713 (51.4%) | 673 (48.6%) | 1386 |
|  | Monthly | 3541 (82.0%) | 779 (18.0%) | 4320 |
|  |  |  |  |  |
| BR | Diarrhea | 80 (87.9%) | 11 (12.1%) | 91 |
|  | Monthly | 2722 (95.6%) | 126 (4.4%) | 2848 |
|  |  |  |  |  |
| IN | Diarrhea | 478 (74.5%) | 164 (25.5%) | 642 |
|  | Monthly | 3994 (83.5%) | 787 (16.5%) | 4781 |
|  |  |  |  |  |
| NP | Diarrhea | 832 (91.8%) | 74 (8.2%) | 906 |
|  | Monthly | 4742 (94.2%) | 296 (5.9%) | 5038 |
|  |  |  |  |  |
| PE | Diarrhea | 1100 (68.5%) | 506 (31.5%) | 1606 |
|  | Monthly | 3187 (76.7%) | 969 (23.3%) | 4156 |
|  |  |  |  |  |
| PK | Diarrhea | 1589 (86.3%) | 252 (13.7%) | 1841 |
|  | Monthly | 4014 (86.9%) | 606 (13.1%) | 4620 |
|  |  |  |  |  |
| SA | Diarrhea | 103 (88.8%) | 13 (11.2%) | 116 |
|  | Monthly | 4095 (89.2%) | 491 (10.7%) | 4586 |
|  |  |  |  |  |
| TZ | Diarrhea | 138 (86.3%) | 22 (13.8%) | 160 |
|  | Monthly | 3893 (91.9%) | 343 (8.1%) | 4236 |

# **Table S2.** Detection of adenovirus 40/41 by qPCR in diarrheal and non-diarrheal (monthly) stools by site and overall, using cycle threshold < 30.

| **Sites** | **Stool type** | **Negative**  **(N=39126)** | **Positive**  **(N=2207)** | **Total**  **(N=41333)** |
| --- | --- | --- | --- | --- |
| Overall | Diarrhea | 6050 (89.7%) | 698 (10.3%) | 6748 |
|  | Monthly | 33076 (95.6%) | 1509 (4.4%) | 34585 |
|  |  |  |  |  |
| BG | Diarrhea | 1107 (79.9%) | 279 (20.1%) | 1386 |
|  | Monthly | 4067 (94.1%) | 253 (5.9%) | 4320 |
|  |  |  |  |  |
| BR | Diarrhea | 86 (94.5%) | 5 (5.5%) | 91 |
|  | Monthly | 2791 (98.0%) | 57 (2.0%) | 2848 |
|  |  |  |  |  |
| IN | Diarrhea | 573 (89.3%) | 69 (10.7%) | 642 |
|  | Monthly | 4537 (94.9%) | 244 (5.1%) | 4781 |
|  |  |  |  |  |
| NP | Diarrhea | 870 (96.0%) | 36 (4.0%) | 906 |
|  | Monthly | 4905 (97.4%) | 133 (2.6%) | 5038 |
|  |  |  |  |  |
| PE | Diarrhea | 1409 (87.7%) | 197 (12.3%) | 1606 |
|  | Monthly | 3852 (92.7%) | 304 (7.3%) | 4156 |
|  |  |  |  |  |
| PK | Diarrhea | 1748 (94.9%) | 93 (5.1%) | 1841 |
|  | Monthly | 4402 (95.3%) | 218 (4.7%) | 4620 |
|  |  |  |  |  |
| SA | Diarrhea | 110 (94.8%) | 6 (5.2%) | 116 |
|  | Monthly | 4469 (97.4%) | 117 (2.6%) | 4586 |
|  |  |  |  |  |
| TZ | Diarrhea | 147 (91.9%) | 13 (8.1%) | 160 |
|  | Monthly | 4053 (95.7%) | 183 (4.3%) | 4236 |
